# Humanitarian Missions: a Call for Action and Impact from Cardiovascular Surgeons

**DOI:** 10.21470/1678-9741-2017-0197

**Published:** 2017

**Authors:** Vinicius José da Silva Nina, Emily A. Farkas, Rachel V. A. H. Nina, Aubyn Marath

**Affiliations:** 1 Universidade Federal do Maranhão (UFMA), São Luís, MA, Brazil.; 2 Member of the Board of Trustees for CardioStart International, Tampa, FL, USA.; 3 Cardiothoracic Surgeon at the ThedaCare Heart Institute, Appleton, WI, USA.; 4 Member of the Board of Trustees for CardioStart International, Emergency USA, Italy and VOOM Foundation, USA.; 5 Universidade Ceuma, São Luís, MA, Brazil.; 6 Volunteer for CardioStart International, Tampa, FL, USA.; 7 Founder and President of CardioStart International, Tampa, USA.

**"Do not wait for leaders; do it alone, person to person. Be faithful in small things because it is in them that your strength lies."**

***Mother Teresa***

***Religious Sister***

Humanitarian aid in the surgical sector is a young science. From before World War II until the late 1980's, little attention was drawn to surgical missions. Throughout that entire period, efforts of international aid agencies were largely directed toward control or eradication of major infectious scourges such as malaria, typhoid, plague, and other tropical and sanitation-based public health problems. It has become evident over the last decade, however, that global epidemiologic and demographic shifts have been changing the burden of disease in all societies. Developing countries are now facing a dramatic increase in noncommunicable diseases, primarily cardiovascular disease (CVD). 2017 Statistics from the World Health Organization confirm that cardiovascular disease remains the leading cause of death globally, and over three quarters (82%) of those CVD deaths take place in low- and middle-income countries^[1]^.

Like Brazil, some of these countries have pockets of excellence in surgical care, but the majority lack such care outside of main cities, amplified by a paucity of necessary equipment and/or formal training. For example, in Germany there is 1 cardiovascular surgeon per 87,000 people, in China there is 1 per 208,000, and in West Africa, there is 1 cardiac surgeon per 26.5 million people^[2]^ (Figure 1).

**Fig. 1:**
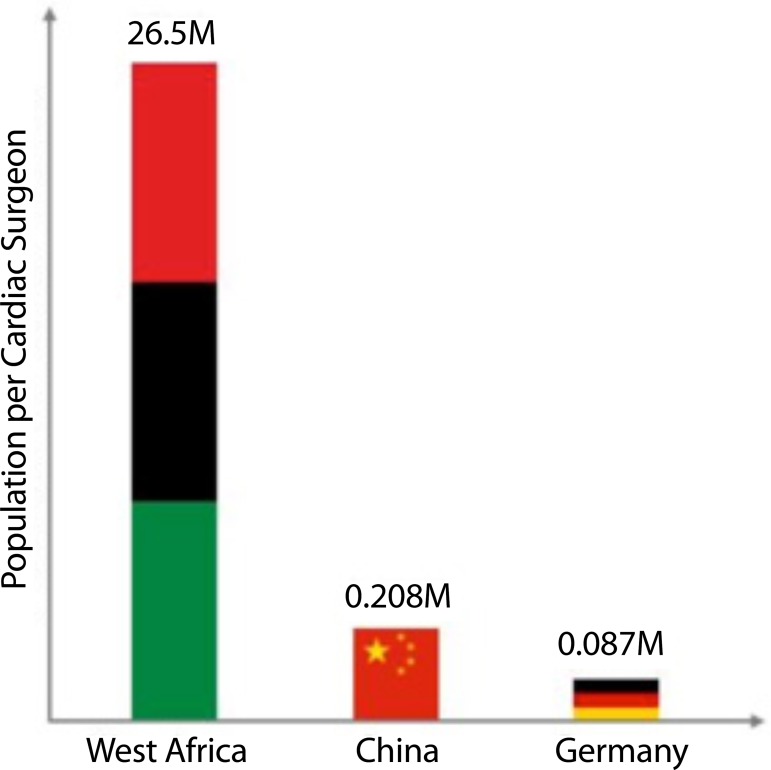
Population in million per cardiac surgeon^[2]^.

Thus, cardiovascular mission formats can be varied, providing preventive care, direct surgical treatment, the donation of supplies and equipment, and/or building a program for sustained training and clinical care. While each objective has value and is interrelated to some extent, every mission follows a different pathway and *modus operandi*^[3]^. We implore surgeons and organizations to craft their missions around this foundation: to train, empower, and support local clinicians toward the shared goal of a self-sustaining center for their community.

While this may seem like an obvious strategy, a common model for many surgical missions surrounds the singular goal of completing as many operations as possible in a short interval. Although this is well intentioned and may serve more patients in the short-term, the scope is myopic, since goals of speed and efficiency often eliminate or minimize teaching, training and the one-to-one skill transfer that is critical for local team advancement. Rather, multiple recurring trips providing graded levels of assistance and support, allowing the local team to take an increasingly primary role in the progression toward independence. Taking the time to teach surgical judgment, intraoperative and postoperative management may limit the number of cases achieved per mission, but is a critical investment in the local clinicians that will pay dividends for the community in the long term.

Hundreds of organizations provide medical and public health services around the world; they include religious brigades, non-governmental organizations (NGOs), relief groups, United Nations affiliates, and military health regiments. Several groups focus on cardiovascular care by trying to reduce the imbalance in access to it across the globe. Humanitarian organizations such as CardioStart International, Team Heart, VOOM Foundation, and Heart to Heart, amongst many others, rely on volunteer teams donating their time and expertise to provide diagnostic investigations and surgical treatment for both acquired and congenital heart defects. Providers are largely from the Western world; however, manpower and local volunteers are keys to the success of any mission^[4,5]^, and it is important to consider the vision and goals of any organization with which one aligns.

To distill it down further, there are many contextual features about medical volunteer work that can provide ethical challenges to volunteers. By the very nature of the work, volunteers travel to areas that lack resources including equipment, personnel, and infrastructure. Thus, there are three major challenges to be faced in preparation for a humanitarian mission: 1) foreign setting; 2) sustainability; and 3) medical education^[6]^.

First, there is an understandable preoccupation with avoiding complications that may arise from working with unfamiliar colleagues in a foreign setting and caring for patients who speak a different language and may have never before seen a doctor. Complications that do occur are often attributable to insufficient screening of patients or inadequate follow-up, so it is of paramount importance to develop strong ties with local physicians who can offer preoperative insight and postoperative continuity^[6,7]^. This is a crucial collaboration at every step, since even surgical decisions such as valve prosthesis often rely on local and/or cultural dynamics, such as whether a patient will be able to afford or have access to warfarin, or if the nearest clinic to check an INR is a 2-day walk in good weather conditions from a remote village.

The second feature is important not only for volunteers, but also for institutions and sponsors of medical service trips to consider logistics and the protocol-implementation required to maintain sustainability within the constructs of the host program. This may involve how their work will advance beyond the short-term experience, training local providers, researching supply chains, establishing local infrastructure, and maintaining long-term relationships. The visiting team must work in collaboration with the local team to build an empowering partnership based on respect for their skills, knowledge, traditions and, whenever possible, using locally available equipment and supplies, such as medications to closely replicate a future scenario sustained without international aid^[5,7]^.

Lastly, the fundamental and primary goal of any mission should be to provide teaching to local staff that encourages methods & techniques to support the improvement of patient care for the long run. This can be achieved most effectively by implementing a long-term educational programme. According to Corno^[8]^, the most suitable and consistent model of long-term humanitarian educational programmes should include the following steps: 1) site selection; 2) demographic research; 3) site assessment; 4) organization of surgical educational teams; 5) regular frequency of surgical educational missions; 6) programme evolution and maturation; 6) educational outreach and interactive support.

Again, this stresses the balance of direct patient care with medical education as very important and emphasizes that any surgical society, organization, institution or individual must prioritize the purpose and motivation for serving. A humanitarian surgeon's focus will always be on patients, but local health care providers and the communities they serve can, and should, both benefit. Ultimately, success should not be measured by the number of successful operations of any given mission, but by the successful operations that the local team performs after the visiting team leaves^[6,8,9]^.

**"The next generation of surgeons, while meeting needs locally, must also take a leadership role globally - the need for international partnership has never been greater."**

***Doruk Ozgediz, MD***

In this context, a volunteer is one who acts in recognition of a need, with an attitude of social responsibility and without concern for monetary profit, gaining beyond what is necessary to one's physical well-being. As a specialty, cardiovascular surgeons are uniquely positioned to have an important role in national/ international, home/abroad, or domestic/foreign humanitarian activity because we are well suited to adapt, improvise and function in unusual or unexpected situations that often require "thinking outside of the box".

The enduring commitment of surgeons to these matters inspires confidence that solutions will continue to come from the surgical community, in keeping with a rich professional legacy. With an emerging generation passionate about their ability to give back in a global society, and so many practicing and retired surgeons pursuing similar opportunities to contribute, the time is ripe to foster these interests and actions^[10]^.

**"By giving of your time and heart, you will not only help to advance the humane practice of surgery, but you will also reap the rewards of belonging to the greatest humanitarian profession in the world."**

***Kathryn Anderson, MD, FACS***

## References

[1] Noncommunicable Diseases Progress Monitor (2017). Geneva: World Health Organization; 2017. Licence: CC BY-NC-SA 3.0
IGO.

[2] Yankah C, Fynn-Thompson F, Antunes M, Edwin F, Yuko-Jowi C, Mendis S (2014). Cardiac surgery capacity in Sub-Saharan Africa: quo
vadis?. Thorac Cardiovasc Surg.

[3] Badlani G (2017). International volunteerism and global
responsibility. Transl Androl Urol.

[4] Wolfberg AJ (2006). Volunteering overseas: lessons from surgical
brigades. N Engl J Med.

[5] Stone GS, Olson KR (2016). The ethics of medical volunteerism. Med Clin North Am.

[6] Maluf MA, Franzoni M, Melgar E, Hernandez A, Perez R (2009). The pediatric cardiac surgery as a philanthropic activity in the
country and humanitarian mission abroad. Rev Bras Cir Cardiovasc.

[7] Dearani JA, Jacobs JP, Bolman 3rd RM, Swain JD, Vricella LA, Weinstein S (2016). Humanitarian outreach in cardiothoracic surgery: from setup to
sustainability. Ann Thorac Surg.

[8] Corno AF (2016). Paediatric and congenital cardiac surgery in emerging economies:
surgical 'safari' versus educational programmes. Interact Cardiovasc Thorac Surg.

[9] Pezzella AT (2006). Volunteerism and humanitarian efforts in surgery. Curr Probl Surg.

[10] Casey KM (2007). The global impact of surgical volunteerism. Surg Clin North Am.

